# *MiR-376c* Down-Regulation Accelerates EGF-Dependent Migration by Targeting *GRB2* in the HuCCT1 Human Intrahepatic Cholangiocarcinoma Cell Line

**DOI:** 10.1371/journal.pone.0069496

**Published:** 2013-07-26

**Authors:** Jun Iwaki, Kunio Kikuchi, Yoshiaki Mizuguchi, Yutaka Kawahigashi, Hiroshi Yoshida, Eiji Uchida, Toshihiro Takizawa

**Affiliations:** 1 Department of Molecular Medicine and Anatomy, Nippon Medical School, Tokyo, Japan; 2 Department of Surgery for Organ Function and Biological Regulation, Nippon Medical School, Tokyo, Japan; Institute of Medical Science, University of Tokyo, Japan

## Abstract

MicroRNA *miR-376c* was expressed in normal intrahepatic biliary epithelial cells (HIBEpiC), but was significantly suppressed in the HuCCT1 intrahepatic cholangiocarcinoma (ICC) cell line. The biological significance of the down-regulation of *miR-376c* in HuCCT1 cells is unknown. We hypothesized that *miR-376c* could function as a tumor suppressor in these cells. To test this hypothesis, we sought the targets of *miR-376c*, and characterized the effect of its down-regulation on HuCCT1 cells. We performed proteomic analysis of *miR-376c*-overexpressing HuCCT1 cells to identify candidate targets of *miR-376c*, and validated these targets by 3′-UTR reporter assay. Transwell migration assays were performed to study the migratory response of HuCCT1 cells to *miR-376c* overexpression. Furthermore, microarrays were used to identify the signaling that were potentially involved in the *miR-376c*-modulated migration of HuCCT1. Finally, we assessed epigenetic changes within the potential promoter region of the *miR-376c* gene in these cells. Proteomic analysis and subsequent validation assays showed that *growth factor receptor-bound protein 2* (*GRB2*) was a direct target of *miR-376c*. The transwell migration assay revealed that *miR-376c* significantly reduced epidermal growth factor (EGF)-dependent cell migration in HuCCT1 cells. DNA microarray and subsequent pathway analysis showed that interleukin 1 beta and matrix metallopeptidase 9 were possible participants in EGF-dependent migration of HuCCT1 cells. Bisulfite sequencing showed higher methylation levels of CpG sites upstream of the *miR-376c* gene in HuCCT1 relative to HIBEpiC cells. Combined treatment with the DNA-demethylating agent 5-aza-2′-deoxycytidine and the histone deacetylase inhibitor trichostatin A significantly upregulated the expression of *miR-376c* in HuCCT1 cells. We revealed that epigenetic repression of *miR-376c* accelerated EGF-dependent cell migration through its target *GRB2* in HuCCT1 cells. These findings suggest that *miR-376c* functions as a tumor suppressor. Since metastasis is the major cause of death in ICC, microRNA manipulation could lead to the development of novel anti-cancer therapy strategies for ICC.

## Introduction

Deep sequencing and transcriptome analysis revealed the existence of non-coding RNAs (ncRNAs) in mammalian cells [1]–[3]. MicroRNAs (miRNAs) are single-stranded 19- to 25-nucleotide ncRNAs that play a critical role in posttranscriptional gene regulation. The miRNA-mediated gene silencing is regulated by complementarity between nucleotides at positions 2–8 of the miRNAs (*i.e.*, the seed sequences) and the 3′-untranslated regions (3′-UTR) of their target genes [4], [5]. Dysregulated expression of miRNA is involved in a variety of human diseases, including cancer and liver disease [6], [7]. Recent studies show that expression levels of various miRNAs are downregulated in cancer, indicating that they may function as tumor suppressors [8]–[10]. For example, downregulation of *miR-15* and *miR-16* in most chronic lymphocytic leukemia cells leads to upregulation of anti-apoptotic B cell lymphoma 2 (Bcl-2) protein [8]. The upregulated Bcl-2 averts apoptotic cell death of leukemia cells and thereby promotes their survival. *Let-7*, which is downregulated in lung cancers relative to normal lung tissue, targets *ras* oncogenes [10]. The increased levels of Ras protein in lung cancer cells leads to upregulated cell growth. The *miR-200* family and *miR-205* target zinc finger homeodomain enhancer-binding protein (ZEB) transcription factors, which are known to be inducers of the epithelial-mesenchymal transition in breast cancer [9]. Downregulation of these miRNAs are likely to be an essential early step in breast cancer metastasis.

Cholangiocarcinoma (CC) is a bile duct cancer, and is classified as intrahepatic or extrahepatic [11]–[13]. Intrahepatic CC (ICC) is derived from epithelial cells of the bile ducts. Although ICCs comprise only 5–10% of all cases of liver cancer, they are the second most common liver malignancy [14]. The incidence and mortality rate of ICC are increasing worldwide. Despite advances in surgical techniques, chemotherapies and radiotherapies, long-term survival remains low because of the late presentation of the disease [14], [15]. Even after resection, the prognosis for patients with advanced ICC is extremely poor [14], [16], [17]. Some researchers have examined the miRNA expression profiles in ICC, to understand the molecular and clinical basis of carcinogenesis and the progression of this disease [18], [19].

We reported previously that *miR-376c* (formerly designated as *miR-368* in miRBase Release 12; currently designated as *miR-376c-3p* in miRBase Release 19) was expressed in a normal intrahepatic biliary epithelial cell line (HIBEpiC), but was significantly suppressed in an ICC cell line (HuCCT1) [18]. However, the biological significance of the downregulation of *miR-376c* in HuCCT1 cells was unknown. We hypothesized that *miR-376c* could function as a tumor suppressor in these cells. To test this hypothesis, we sought the targets of this miRNA, and characterized the effect of *miR-376c* down-regulation in HuCCT1 cells. We identified a direct target mRNA of *miR-376c* by proteomic analysis. Enforced expression of *miR-376c* significantly impaired migration of HuCCT1 cells by reducing levels of the targeted mRNA, indicating that downregulation of *miR-376c* is critical for this response. Finally, we investigated the DNA methylation and histone modification status of the putative promoter regions of *miR-376c* gene in HuCCT1 cells.

## Materials and Methods

### Cell culture and RNA isolation

HIBEpiC were purchased from ScienCell Research Laboratories (Carlsbad, CA, USA). HuCCT1 was obtained from the American Type Culture Collection (Manassas, VA, USA). The ICC cell lines TKKK, Huh28, and IHGGK, and the extrahepatic CC cell line TFK-1 were from RIKEN BioResource Center (Tsukuba, Japan). All cell lines were maintained in the media recommended by the suppliers, at 37°C in a humidified incubator with a 5% CO_2_ atmosphere. Total RNA was extracted from each sample using RNAiso plus (Takara Bio, Ohtsu, Japan), according to the manufacturer's instructions.

### Proteomic analysis

Proteomic analysis was performed based on our previous method [20]. HuCCT1 cells grown to semi-confluence were transfected with Pre-miR-376c (mature *miR-376c-3p* mimic) or Pre-miR negative control #1 (Applied Biosystems, Foster City, CA, USA) at a final concentration of 30 nM using Lipofectamine 2000 (Invitrogen, Carlsbad, CA, USA), according to the manufacturer's protocol. Pre-miR-transfected HuCCT1 cells were harvested 72 h after transfection and subjected to the following proteomic analysis. Cells were lysed with a thiourea lysis buffer (7.5 M urea, 2 M thiourea, 4% 3-[(3-cholamidopropyl) dimethylammonio]-1-propanesulfonate, 1 mM PMSF, 1 µM aprotinin, 1 µM pepstatin A, and 10 mM Tris-HCl [pH 8.8]). After removal of cell debris by centrifugation at 20,000× *g* at 4°C for 20 min, proteins in the cell lysates were labeled with CyDye DIGE Fluors minimal dyes (GE Healthcare Bio-Sciences, Little Chalfont, UK). The labeled proteins were subjected to two-dimensional difference gel electrophoresis (2D-DIGE) using the Ettan DALTsix Large Electrophoresis System (GE Healthcare Bio-Sciences), as described in the manufacturer's protocol. Protein samples obtained from three independent experiments were subjected to proteomic analysis. Spots with significant differences in intensity between the samples (>20% upregulation or downregulation, *P*≤0.011) were excised from the gel using the Ettan Spot Picker (GE Healthcare Bio-Sciences). These criteria for spot selection were determined based on previous studies, in which the repression of cellular protein synthesis by miRNA overexpression was typically mild [21], [22]. Proteins in the spots were subjected to digestion with 10 ng/µl trypsin (Sigma, St. Louis, MO, USA) at 37°C, followed by elution in an acetonitrile buffer. The eluted proteins were then identified using a liquid chromatography-tandem mass spectrometry mass spectrometer (LCQ DECA XP Plus; Thermo-Finnigan, San Jose, CA, USA). Mass spectrometry data were analyzed using the MASCOT software (Matrix Science, London, England). Specificity of trypsin digestion was used as the cutting enzyme with allowing three missed cleavages. Peptide mass tolerance and fragment mass tolerance were set to ±1.2 Da and ±0.6 Da, respectively. Carbamidomethyl of cysteine was chosen as the fixed modification and oxidation of methionine was searched as the variable modification. Identity or extensive homology is indicated for peptides with individual ion scores >42 (*p*<0.05).

### Western blotting

HuCCT1 cells in culture were rinsed in PBS and lysed in M-PER Mammalian Protein Extraction Reagent (Thermo Fisher Scientific, Rockford, lL, USA) containing Halt Protease Inhibitor Cocktail (Thermo Fisher Scientific). The lysates were centrifuged at 20,000× *g* at 4°C for 3 min to remove debris, and supernatants were subjected to western blotting as follows: the lysates were separated on SDS-PAGE gels and transferred to Sequi-Blot polyvinylidene fluoride membranes (Bio-Rad, Hercules, CA, USA). Blots were then incubated at 4°C overnight with primary antibodies diluted as recommended in the manufacturer's instructions. This was followed by incubation with horseradish peroxidase-conjugated secondary antibodies. Signals were detected using Immobilon reagent (Millipore, Billerica, MA, USA) and visualized using an LAS-4000 Lumino image analyzer (Fujifilm, Tokyo, Japan). The intensity of visualized signals was quantitated using the Multigauge software (Fujifilm). A monoclonal GRB2 antibody (catalog number 61011) was purchased from Becton Dickinson (Franklin Lakes, NJ, USA). A monoclonal antibody for beta actin (ACTB) was purchased from Sigma (catalog number A5316).

### Real-time PCR

Real-time PCR for miRNAs was carried out using TaqMan Gene Expression Assays (Applied Biosystems) in a 7300 Real-Time PCR System (Applied Biosystems) or a 7900 FAST Real-Time PCR System (Applied Biosystems), according to the manufacturer's protocols. *U6* snRNA (*RNU6B*) was used as an endogenous internal control. To quantify mRNA levels, SYBR Premix Ex Taq (Perfect Real Time) (Takara Bio) was applied. To normalize mRNA expression levels, *glyceraldehyde 3-phosphate dehydrogenase* (*GAPDH*) was used as an endogenous internal control. Primers used for RT-PCR of *GRB2*, *IL1B*, *MMP9* were as follows: *GRB2*-Foward: CCATCGCCAAATATGACTTCAAA; *GRB2*-Reverse: CTTCGTTCAAAACCTTGAGGATGT; *IL1B* -Forward: CAACAAGTGGTGTTCTCCATGT; *IL1B*-Reverse: GACAAATCGCTTTTCCATCTTC; *MMP9*-Foward: ACTTTGACAGCGACAAGAGGTGG; *MMP9*-Reverse: CCGGCACTGAGGAATGATCTAA.

### 3′-UTR reporter assay

To construct a reporter plasmid, we first cloned the 3′-UTR of the human *GRB2* into pMIR-REPORT vector (Applied Biosystems). Total RNA, isolated from HuCCT1 cells, was reverse-transcribed to cDNA using PrimeScript reverse transcriptase (Takara Bio). The 3′-UTR of *GRB2* mRNA was then amplified from the cDNA using the following primers: GAACTAGTGAGTCAAGAAGCAATTATTTAAAGAAAGTGAA (*Spe*I site underlined) and GAAAGCTTTGAAGAATTCATTGTGTATTTATTATTCACAG (*Hind*III site underlined). The PCR product was cloned into a pCR4-Blunt-TOPO vector for sequence verification. The *GRB2* 3′-UTR was then cloned into pMIR-REPORT via the *Spe*I and *Hind*III restriction sites. This final construct was designated pMIR-GRB2. To construct a reporter plasmid with a mutated *miR-376*c recognition site of *GRB2* 3′-UTR, an inverse PCR method was used. The primers used for the inverse PCR were: *GRB2* 3′UTRmutinv-Foward: ggtattcgtatctctcaaaaTGTCTGTTTTAGTTGGAATG; *GRB2* 3′UTRmutinv-Reverse: ttttgagagatacgaataccAGCCCACTTGGTTTCTTGTT (complementary sequence shown in lowercase), and were designed to introduce the mutation. PCR amplification was carried out using the previously cloned vector pCR4-Blunt-TOPO containing the *GRB2* 3′-UTR. Plasmid DNA was digested by *Dpn*I. Amplified DNA was transformed into *Escherichia coli*. After sequence verification, the mutated 3′-UTR sequence was cloned into pMIR-REPORT via *Spe*I and *Hind*III restriction sites. The final construct was designated pMIR-GRB2mt. HuCCT1 cells were transfected with the pMIR-GRB2, pMIR-GRB2mt, or the control vector pRL-TK (Renilla luciferase expression plasmid), together with Pre-miR-376c or the Pre-miR-negative control at 10 nM, using Lipofectamine 2000 (Invitrogen, Carlsbad, CA, USA) in 24-well plates. Twenty-four hours after transfection, luciferase assays were performed using the Dual Luciferase Reporter Assay System (Promega, Madison, WI, USA). Firefly luciferase activity was normalized to Renilla luciferase activity.

### Cell migration assay

HuCCT1 cell migration analysis was carried out using Transwell inserts (8.0 µm pore size, Costar, Cambridge, MA, USA) [23]. Cells were transfected with the Pre-miR molecule or *GRB2*-siRNA at final concentrations of 30 nM using Lipofectamine 2000 (Invitrogen). Two siRNAs for *GRB2*, siGRB2-1 (Hs_GRB2_5; sense: GUUUGGAAACGAUGUGCAGTT; antisense: CUGCACAUCGUUUCCAAACTT) and siGRB2-2 (Hs_GRB2_8; sense: AGAACUACAUAGAAAUGAATT; antisense UUCAUUUCUAUGUAGUUCUTG) were used (Qiagen, Valencia, CA, USA). A nonspecific non-silencing siRNA (AllStars Negative Control siRNA, Qiagen) was used as a negative control. After 24 h of transfection, cells (5×10^4^) in 250 µl of serum-free medium RPMI 1640 were seeded onto filters in 24-well plates. The medium (750 µl) containing RPMI 1640 supplemented with 0.1% fetal bovine serum was placed in the lower chamber in the presence of epidermal growth factor (EGF) (5 ng/ml, R&D Systems, Minneapolis, USA). After incubation for 24 h, non-invading cells on the top of each Transwell were scraped off with a cotton swab. Cells that had migrated to the other side were fixed with 2.5% glutaraldehyde (Wako, Tokyo, Japan) and stained with crystal violet (Wako). The number of migrated cells was manually counted with a light microscope (KX4, Olympus, Tokyo, Japan) under 200× magnification. The sum of the numbers of cells in five areas was used as the migrated cell number, and expressed as a percentage of the control value. These experiments were repeated at least three times, and significant differences among treatments were assessed by ANOVA followed by Tukey's test. In our preliminary experiments of cells exposed to different concentrations of EGF (0.1, 0.5, 1, 5, and 10 ng/ml), EGF regulation of cell migration was in a dose-dependent manner. HuCCT1 cell migration peaked at the 5 ng/ml concentration but then decreased at 10 ng/ml (data not shown). The preliminary experiments indicate that the optimized concentration of EGF for cell migration assay was 5 ng/ml.

### Gene expression analysis

HuCCT1 cells were treated with EGF as described above. Total RNA was extracted from Pre-miR-376c*-* and siGRB2*-*2-transfected HuCCT1 cells and from individual transfection controls with RNAiso Plus (Takara Bio), and its concentration was measured using a NanoDrop ND-2000 spectrometer (Thermo Scientific, Wilmington, DE, USA). Of the total RNA obtained, 200 ng was used in a labeling reaction, using a Low-Input QuickAmp Labeling Kit, One-Color (Agilent Technologies, Santa Clara, CA, USA), and the quality and yield of labeled cRNA was evaluated on an Agilent 2100 Bioanalyzer (Agilent). Gene expression profiling was conducted using an Agilent microarray (Human GE 4×44 K, v2). The resulting signals were normalized to the 75^th^ percentile signal intensity and the processed data were filtered for an at least twofold change using the GeneSpring GX software (*ver.* 11.5; Agilent). The array data were imported into the Ingenuity Pathway Analysis software (IPA, Ingenuity Systems, Redwood City, CA, USA). Functional analyses of down-regulated genes were conducted using the IPA software (Ingenuity Systems, http://www.ingenuity.com). A right-tailed Fisher's exact test was used to calculate a *p*-value for the probability of an association between the gene expression data and the knowledge-based biological function. The mRNA array data are publicly available (Gene Expression Omnibus accession number GSE47186, http://www.ncbi.nlm.nih.gov/geo/).

### Bisulfite conversion of DNA and bisulfite sequencing

Genomic DNA was extracted using a DNA pure kit (Takara Bio), according to the manufacturer's protocol. Bisulfite conversion of DNA (1 µg) was performed using a Methyl Easy Xceed Rapid DNA modification kit (Takara Bio) according to the manufacturer's protocol. PCR amplification was performed using Ex Taq HS (Takara Bio). Primer sequences were TTGAATATTTTTGAGAGGAAGGTTAGT and TCCTAAAAAACATAAACCTAAACACAAT. PCR products were ligated into pCR4 TOPO (Invitrogen). At least 20 clones were sequenced per cell.

### Treatment with 5-AZA-dCR and TSA

HuCCT1 cells were seeded at a density of 5×10^4^/well in six-well plates, cultured for 24 h and then treated with 10 µM DNA-demethylating agent 5-aza-2′-deoxycytidine (5-AZA-dCR, Sigma-Aldrich, St. Louis, MO, USA) for 3 days, during which the drug-containing medium was replaced daily. After 3 days, the histone deacetylase (HDAC) inhibitor trichostatin A (TSA, Sigma-Aldrich; 0.1, 0.5, or 1.0 µM) was added and cells were cultured for a further 24 h.

## Results

### *MiR-376c* is downregulated in CC cell lines including HuCCT1

**Figure 1:**
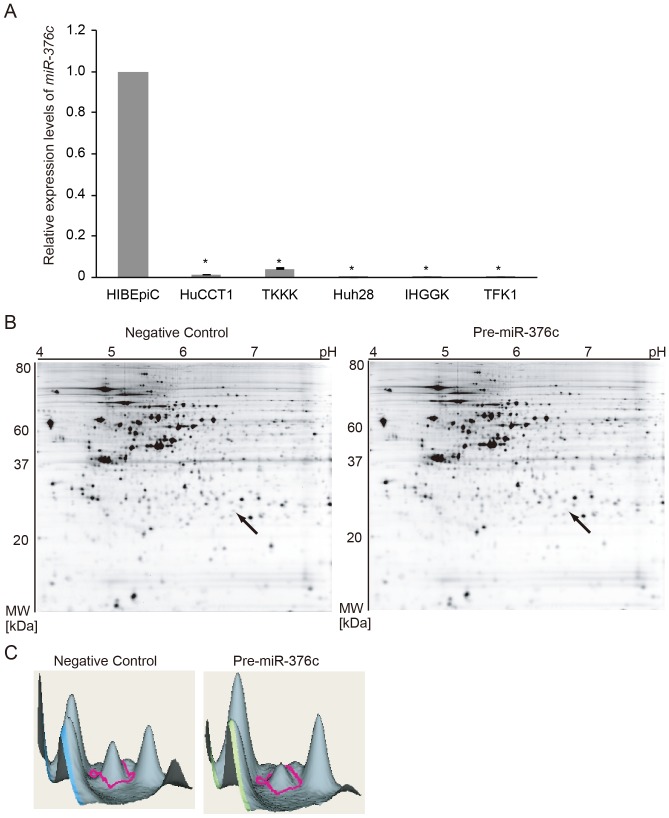
Downregulation of *miR-376c* expression levels in bile duct carcinoma cell lines, and proteomic analysis of *miR-376c*-overexpressing HuCCT1. (**A**) Real-time PCR assay of *miR-376c* in HIBEpiC, HuCCT1, Huh28, IHGGK, TKKK, and TFK1. Expression levels were normalized to *RNU6B*, and the expression level in HIBEpiC cells was defined as 1. The significance of differences among cells was assessed by ANOVA followed by Tukey's test (*P<0.05). (**B**) Representative 2D-DIGE images of *miR-376c*-overexpressing HuCCT1 cells. Cells were harvested 72 h after the initiation of transfection of Pre-miR-376c or the Pre-miR-negative control, and subjected to proteomic analysis. A spot downregulated by treatment with Pre-miR-376c is indicated by the arrow, which was later shown by mass spectrometry to be GRB2. (**C**) Quantitative analysis of the fluorescence intensity of the GRB2 protein spot shown in **B** (peak outlined in red).

We previously performed small RNA sequencing using HuCCT1 and HIBEpiC to reveal differential miRNA expression [18]. Small RNA sequencing showed that *miR-376c* was found exclusively in HIBEpiC and not in HuCCT1 (*i.e.*, cloning frequency >0.1% in HIBEpiC and 0.0% in HuCCT1) [18]. For further characterization of *miR-376c* expression in CC cell lines, the ICC cell lines TKKK, Huh28, and IHGGK, and the extrahepatic CC cell line TFK-1 were examined. Real-time PCR analysis revealed that *miR-376c*, which was highly expressed in HIBEpiC, was significantly downregulated in all CC cell lines examined in this study (Figure 1A). It is likely that *miR-376c* is downregulated specifically in bile duct carcinoma cell lines.

### *MiR-376c* target identification by proteomic analysis

We performed a proteomic analysis to identify proteins whose expression was altered in *miR-376c*-overexpressing HuCCT1 cells, some of which may be targets of *miR-376c*. Proteomic analysis was performed by 2D-DIGE (Figure 1B). We found 41 protein spots with significant differences in intensity between controls and Pre-miR-376c-transfected HuCCT1 cells (upregulation or downregulation by more than 1.2-fold, *P*≤0.011). Of these 41 spots, 18 and 31 spots exhibited upregulation and downregulation of intensity, respectively. These spots were isolated and subjected to liquid chromatography-tandem mass spectrometry analysis. We focused on the downregulated proteins as potential direct targets of *miR-376c* (Table 1). Using the online software TargetScan (http://www.targetscan.org) and miRbase (http://mirbase.com), we identified only GRB2 protein as an *in silico* candidate; its expression level was decreased by 44% (Table 1 and Figure 1C).

**Table 1:** Proteins downregulated by *miR-376c* overexpression in HuCCT1 cells in proteomic analysis.*

FC	*P*-value	Protein Name	Gene Name
−2.22	0.011	cytoskeleton-associated protein 4	*CKAP4*
		Alpha-2-HS-glycoprotein precursor	*AHSG*
−2.21	0.011	cofilin 1 (non-muscle)	*CFL1*
−1.85	0.0027	lamin A/C isoform 1 precursor	*LMNA*
		ERO1-like	*ERO1L*
		leukotriene A4 hydrolase	*LTA4H*
		Alpha-2-HS-glycoprotein precursor	*AHSG*
		very-long-chain acyl-CoA dehydrogenase	*ACADVL*
		80K-H protein, protein kinase C substrate 80K-H isoform 1	*G19P1*
−1.58	0.0097	calpain, small subunit 1	*CAPNS1*
−1.52	0.0086	Keratin, type I cytoskeletal 19	*KRT19*
−1.51	0.0092	lamin A/C isoform 2	*LMNA*
		ERO1-like	*ERO1L*
		very-long-chain acyl-CoA dehydrogenase	*ACADVL*
		heterogeneous nuclear ribonucleoprotein L	*HNRPL*
		tubulin 5-beta	*TUBB4*
−1.51	0.01	transferase, HG phosphoribosyl	*HPRT*
−1.48	0.0033	cathepsin D preproprotein	*CTSD*
		splicing factor, arginine/serine-rich 9	*SFRS9*
−1.44	0.0094	**growth factor receptor-bound protein 2 isoform 1**	***GRB2***
		Ras family small GTP binding protein TC21	*RRAS2*
−1.42	0.0024	Annexin A2	*ANXA2*
		glyceraldehyde-3-phosphate dehydrogenase	*GAPDH*
		serine dehydratase-like	*SDSL*
−1.42	0.006	annexin A2 isoform 2	*ANXA2*
		glyceraldehyde-3-phosphate dehydrogenase	*GAPDH*
		ribosomal protein P0	*RPLP0*
		F-actin capping protein beta subunit	*CAPZB*
		capping protein alpha	*CAPZA2*
−1.4	0.0062	LASP1 protein	*LASP1*
		heterogeneous nuclear ribonucleoprotein D-like	*HNRPDL*
		annexin A2 isoform 2	*ANXA2*
−1.39	0.0097	Annexin A2	*ANXA2*
		glyceraldehyde-3-phosphate dehydrogenase	*GAPDH*
		ribosomal protein P0	*RPLP0*
−1.35	0.0041	cytokeratin 8 (279 AA)	*KRT8*
		tyrosine 3/tryptophan 5 -monooxygenase activation protein, zeta polypeptide	*YWHAZ*
		GRP78 precursor, BiP protein	
		tumor protein D52-like 2 isoform a	*TPD52L2*
		proteasome alpha 3 subunit isoform 1	*PSMA3*
		rho GDP dissociation inhibitor (GDI)	*ARHGDIA*
−1.35	0.0044	cathepsin D preproprotein	*CTSD*
		mitochondrial ribosomal protein L46	*MRPL46*
		platelet-activating factor acetylhydrolase, isoform Ib, beta subunit 30 kDa	*PAFAH1B2*
−1.35	0.011	ERO1-like	*ERO1L*
		Ras-GTPase-activating protein SH3-domain-binding protein	*G3BP1*
		lamin A/C	*LMNA*
−1.34	0.0065	cathepsin D preproprotein	*CTSD*
		eukryotic translation elongation factor 1 gamma, isoform CRA a	*EEF1G*
−1.32	0.0061	myosin light chain 3	*MYL3*
		ATP synthase, H+ transporting, mitochondrial F1 complex, delta subunit precursor	*ATP5D*
−1.31	0.0017	Annexin A2	*ANXA2, ANXA2P1*
		glyceraldehyde-3-phosphate dehydrogenase	*GAPDH*
		heterogeneous nuclear ribonucleoprotein D-like	*HNRPDL*
		fibrillarin	*FBL*
		torsin family 1, member A (torsin A)	*TOR1A*
		palmitoyl-protein thioesterase 1	*PPT1*
−1.28	0.002	apolipoprotein L2	*APOL2*
		mitochondria import inner membrane translocase subunit TIM50 precursor	*TIMM50*
		Annexin A2	*ANXA2*
		heterogeneous nuclear ribonucleoprotein D-like	*HNRPDL*
		glyceraldehyde-3-phosphate dehydrogenase	*GAPDH*
−1.26	0.0034	annexin A2 isoform 2	*ANXA2*
		carboxyl terminal LIM domain protein	*CLIM1*
		mitochondrial ribosomal protein L39 isoform b	*MRPL39*
		aflatoxin aldehyde reductase AFAR	*AKR7A2*
		glyceraldehyde-3-phosphate dehydrogenase	*GAPDH*
−1.23	0.0025	keratin	
−1.21	0.0023	ERO1-like	*ERO1L*
		lamin A/C isoform 2	*LMNA*
		chaperonin containing TCP1, subunit 5 (epsilon)	*CCT5*
		Beta-galactosidase precursor	*GLB1*
		heterogeneous nuclear ribonucleoprotein K isoform a	*HNRPK*
		copine I	*CPNE1*
		Ras-GTPase-activating protein SH3-domain-binding protein	*G3BP1*
		DHX9 protein	*DHX9*

* The protein spots that had significant differences in intensity between the control- and Pre-miR-376c-transfected HuCCT1 cells (down-regulation by more than 1.2-fold change, *p*≤0.011) are listed in proteomic analysis. FC: fold change. The GRB2 protein predicted by TargetScan as a target of *miR-376c* is shown in bold.

### Validation of *GRB2* as a *miR-376c* Target

**Figure 2:**
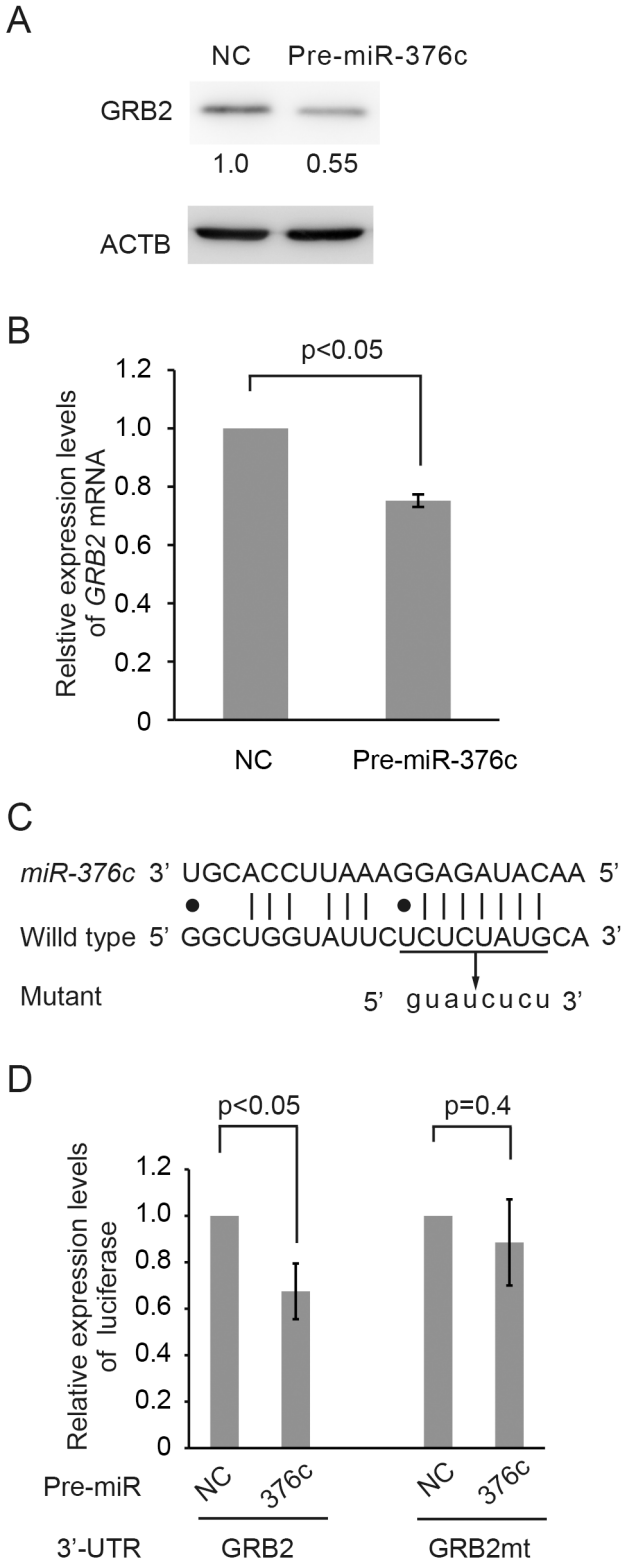
Validation of *GRB2* as a *miR-376c* Target. (**A**) Western blotting analysis of GRB2 protein levels in HuCCT1 cells transfected with Pre-miR-376c or the Pre-miR negative control (NC). ACTB was monitored as an internal control. Relative GRB2 expression levels were calculated and are indicated below the bands. (**B**) *GRB2* mRNA expression levels in HuCCT1 cells transfected with Pre-miR-376c or the Pre-miR negative control (NC). The *GRB2* expression level was normalized to *GAPDH*. The expression level of the NC sample was defined as 1. The significance of differences between means was determined by Student's *t*-test. (**C**) The sequences of the mature *miR-376c* and its putative target site in the 3'-UTR of *GRB2*. The target site corresponding to the seed sequence of *miR-376c* was converted via mutation; the mutation introduced into the *miR-376c* recognition site of *GRB2* 3'-UTR in the reporter plasmid is also shown. (**D**) *GRB2* 3'-UTR luciferase reporter assay. Reporter vector (pMIR-GRB2 [GRB2] or pMIR-GRB2mt [GRB2mt]) and Pre-miR molecule (Pre-miR-376c [376c] or Pre-miR negative control [NC]) were co-transfected into HuCCT1 cells. Renilla luciferase vector pRL-TK was used as an internal control. Luciferase expression levels of Pre-miR negative control (NC) were set to 1.0. The significance of differences between means was determined by Student's *t*-test.

To confirm the 2D-DIGE results, we performed Western blotting analysis of HuCCT1 transfected with Pre-miR-376c. As shown in Figure 2A, the GRB2 protein level was markedly decreased in Pre-miR-376c-transfected cells compared to control cells. Furthermore, as shown in Figure 2B, the *GRB2* mRNA level was also significantly decreased by Pre-miR-376c overexpression.

Next, to determine whether the 3′-UTR of *GRB2* is a direct target of *miR-376c*, we performed a luciferase reporter assay. As shown in Figure 2D, Pre-miR-376c overexpression decreased significantly the luciferase activity in HuCCT1 cells cotransfected with pMIR-GRB2 (by ∼30% compared to the negative control), but not significantly in cells cotransfected with pMIR-GRB2mt, a reporter plasmid mutated in the putative *miR-376c* recognition site of the *GRB2* 3′-UTR (Figure 2C). Taken together, these results suggest that *GRB2* is a target of *miR-376c* in HuCCT1 cells.

### *MiR-376c* regulates epidermal growth factor (EGF)-dependent migration of HuCCT1 via GRB2

**Figure 3:**
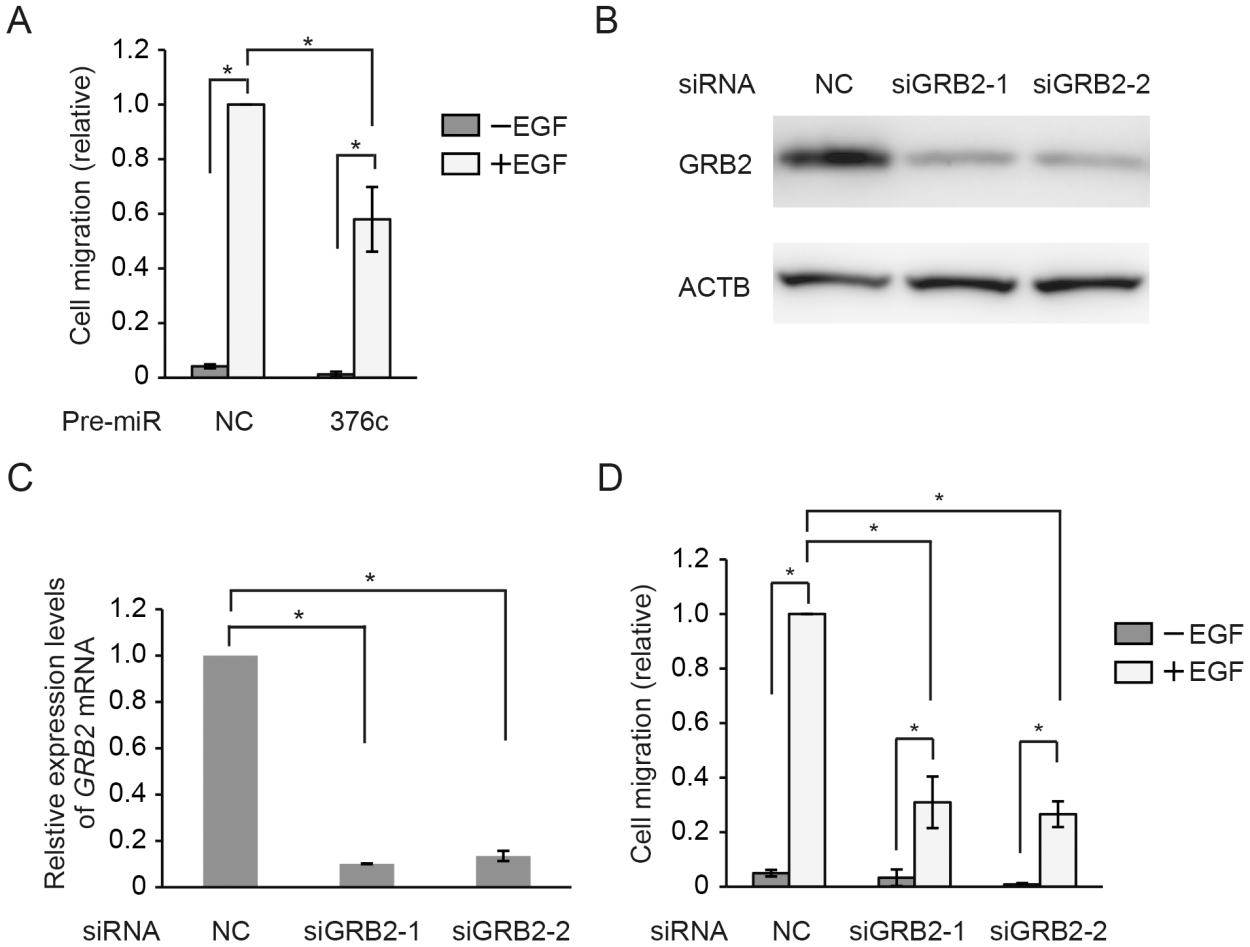
*MiR-376c* represses cell migration via *GRB2* reduction. (**A**) Transwell migration assay of HuCCT1 cells transfected with Pre-miR-376c. Medium containing 5 ng/ml EGF in the lower chamber served as a chemoattractant. After 24 h of transfection, migrating cells were stained and counted. Data are presented as the ratio of the number of migrating Pre-miR-376c (376c)-transfected cells to that of cells transfected with the Pre-miR-negative control (NC), in the presence or absence of EGF. Cell migration of NC in the presence of EGF was set to 1.0. The significance of differences among treatments was assessed by ANOVA followed by Tukey's test (* *p*<0.05). (**B**) Western blotting analysis of the GRB2 protein level in HuCCT1 cells transfected with the siRNAs. Two siRNA molecules targeting *GRB2* (siGRB2-1 and siGRB2-2) and negative control siRNA (NC) were used. ACTB was used as an internal control. (**C**) Real-time PCR analysis of the *GRB2* mRNA level in HuCCT1 cells transfected with the siRNAs. The expression level of cells transfected with negative control siRNA (NC) was set to 1.0. The *GRB2* expression levels were normalized to *GAPDH*. (**D**) Transwell migration assay of HuCCT1 cells transfected with the siRNAs. Data are presented as numbers of migrating siRNA-transfected cells relative to cells transfected with the negative control siRNA (NC), in the presence or absence of EGF. Migration of the negative controls in the presence of EGF was set to 1.0. The significance of differences among treatments was assessed by ANOVA followed by Tukey's test (* *p*<0.05).

GRB2 is known to be a key molecule in intracellular signal transduction through the EGF receptor (EGFR), and can be overexpressed in tumors of breast and bladder cancers [24]–[26]. Molecules that bind to GRB2 protein and inhibit signal transduction have been demonstrated to reduce motility *in vitro* and to decrease cancer metastasis in animal models [24], [27]–[29]. GRB2 plays a critical role in EGFR signal transmission and internalization [30]–[32]. EGF stimulation increases motility in multiple cell lines [32]. Therefore, we assessed the effect of *miR-376c* on migration of HuCCT1 cells stimulated with EGF in transwell assays. In the presence of EGF, the migration of HuCCT1 cells transfected with the Pre-miR-negative control was induced (Figure 3A), indicating that such migration is EGF dependent. Treatment with Pre-miR-376c significantly decreased EGF-induced migration (to 57% of the control value, Figure 3A). Furthermore, to confirm whether EGF-induced migration was dependent on GRB2, we analyzed migration by cells in which *GRB2* was downregulated by siRNA-mediated knockdown. We used two siRNA duplexes specific for *GRB2* (designated siGRB2-1 and siGRB2-2). First, we evaluated the efficiency of siRNA-mediated knockdown by Western blotting and real-time PCR. In HuCCT1 cells, siGRB2-1 and siGRB2-2 inhibited expression of both *GRB2* protein (Figure 3B) and mRNA (Figure 3C). Next, we assayed migration of HuCCT1 cells treated with siRNAs against *GRB2*. After *GRB2* knockdown, EGF-dependent cell migration was reduced significantly (Figure 3D). These results suggest that *miR-376c* regulates EGF-dependent cell migration through repression of *GRB2* translation.

In addition, we assessed the effect of Pre-miR-376c and siGRB2 transfection on cell growth (Figure S1). No significant differences in cell growth were identified between transfected and non-transfected cells. This indicates that GRB2 modulates migration but not growth of HuCCT1 cells.

### Network analysis relevant to GRB2-mediated HuCCT1 migration

**Figure 4:**
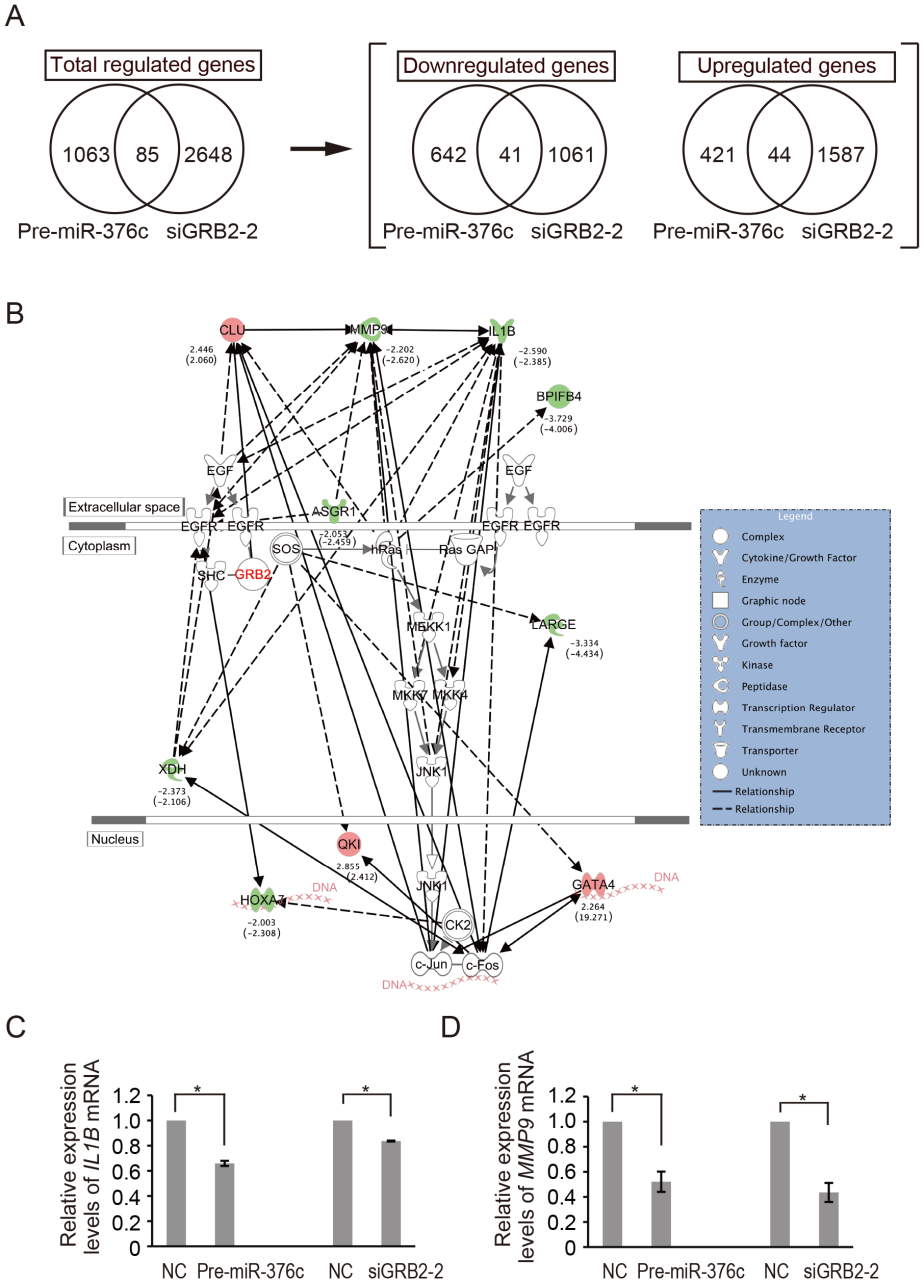
Network analysis relevant to GRB2-mediated HuCCT1 migration. (**A**) Venn diagrams showing the significantly different mRNA expression levels of Pre-miR-376c and siGRB2-2 transfectants of HuCCT1 relative to appropriate controls. Expression profiles of mRNAs affected by Pre-miR-376c and siGRB2-2 in EGF-treated HuCCT1 cells were conducted by microarray analysis. The numbers of genes regulated by Pre-miR-376c and siGRB2-2 are indicated. (**B**) The network of the identified molecules regulated by both Pre-miR-376c and siGRB2-2 in this study were connected with EGF, EGFR and GRB2 by IPA analysis. Numbers below the upregulation (red) and downregulation (green) symbols represent the fold changes by Pre-miR-376c treatment; numbers in parentheses represent the fold changes by siGRB2-2 treatment. Solid and dotted lines indicate direct and indirect gene relationships, respectively. (**C and D**) Real-time PCR analysis of *IL1B* (**C**) and *MMP9* (**D**) expression levels in EGF-treated HuCCT1 cells after transfection with Pre-miR-376c and siGRB2-2. Pre-miR-negative control and nonspecific non-silencing siRNA were used as negative controls (NC). For quantitative comparisons, expression levels were normalized to *GAPDH*. The expression levels in negative controls were set to 1.0. The significance of differences between means was determined by Student's *t*-test. * *p*<0.05.

To elucidate key molecules in EGF-dependent HuCCT1 migration, in which GRB2 upregulation is caused by abnormal suppression of *miR-376c*, we compared expression profiles of mRNAs affected by Pre-miR-376c and siGRB2 (siGRB2-2) in EGF-treated HuCCT1 cells. Microarray analysis showed that changes in expression of 1148 and 2733 genes were induced by Pre-miR-376c and siGRB2-2, respectively. In the microarray data, 85 genes were common to both sets (Figure 4A). Of these, 41 genes were downregulated by both treatments, and 44 were upregulated by both treatments (Table S1 and S2). We next conducted a network pathway analysis, using Ingenuity Pathway Analysis (IPA) to identify molecules relevant to GRB2-mediated cell migration. A network of 85 genes derived from the microarray data with c-Jun N-terminal kinase 1 (JNK1, also known as MAPK8)-mediated EGFR signaling was generated (Figure 4B). Ten of the 85 genes were associated with the EGFR pathway. From the viewpoint of the cellular function of *miR-376c*, two down-regulated genes; *i.e.*, *interleukin-1 beta* (*IL1B*) and *matrix metallopeptidase 9* (*MMP9*), were associated primarily with several cellular-movement networks in the Ingenuity Knowledge Base (Table S3). These data suggest that *IL1B* and *MMP9* function downstream of GRB2 signaling in EGF-dependent cell migration by HuCCT1. Thus, we evaluated the gene suppression of *IL1B* and *MMP9* in HuCCT1 cells treated with Pre-miR-376c and siGRB2-2. Pre-miR-376c significantly decreased (to 66%) expression of *IL1B* (Figure 4C) and *MMP9* (to 52%, Figure 4D), compared to Pre-miR-negative controls. Use of siGRB2-2 also resulted in significant reductions in the expression of these genes, to 83% and 43% relative to controls, respectively (Figure 4C and 4D).

### *MiR-376c* expression is regulated by epigenetic control in HuCCT1

**Figure 5:**
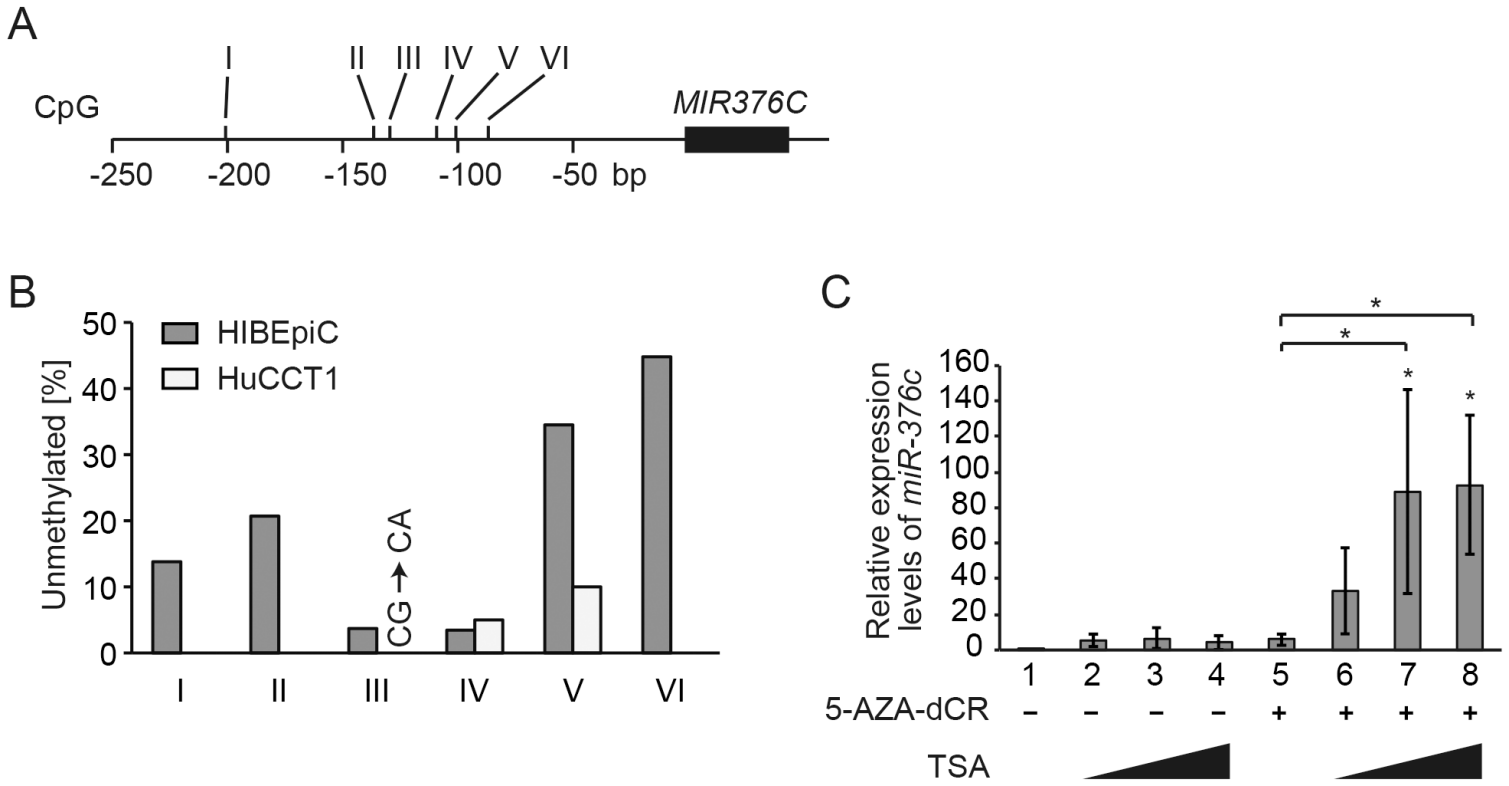
Methylation of *miR-376c*. (**A**) Location of the six CpG sites (sites I–VI) upstream of *miR-376c*, in the putative promoter region of the gene. (**B**) Bisulfite sequencing analysis of these CpG sites in HIBEpiC and HuCCT1. The unmethylated levels of the six sites were expressed as percentages of methylation reference values. A mutation in the genome sequence of HuCCT1 was found at CpG site III. (**C**) Real-time PCR analysis of *miR-376c* expression levels in HuCCT1 cells treated with the DNA-demethylating agent 5-AZA-dCR and/or the HDAC inhibitor TSA. After treatment with 10 µM of 5-AZA-dCR for 3 days, HuCCT1 cells were incubated with TSA (0.1, 0.5, or 1.0 µM) for a further 24 h. Expression levels were normalized to *RNU6B*. The expression level in untreated HuCCT1 cells was defined as 1 (lane 1). Differences among treatments were tested by ANOVA followed by Tukey's test (* *p*<0.05).

To determine whether *miR-376c* expression was dysregulated by epigenetic alterations in HuCCT1 cells, we first assessed the methylation status of the 6 CpG sites (sites I–VI) located 85 to 202 bp upstream of *miR-376c*; *i.e.*, in the putative promoter region, in HIBEpiC and HuCCT1 cells by bisulfite genome sequencing (Figure 5A). A mutation in CpG site III was found in the genome sequence of HuCCT1. We quantified the relative level of methylation of the remaining CpG sites in HIBEpiC and HuCCT1 (Figure 5B). Three sites (I, II, and VI) in HuCCT1 cells were fully methylated, and the unmethylated levels at sites IV and V were 5% and 10%, respectively (Figure 5B). In contrast, in HIBEpiC cells the nonmethylation levels of sites I–VI were 14, 21, 4, 3.5, 35, and 45%, respectively (Figure 5B). These bisulfite sequencing data show that, with the exception of site IV, the levels of methylation at CpG sites in HuCCT1 cells are higher than those in HIBEpiC cells.

DNA methylation and histone acetylation play critical roles in the expression of miRNA genes [33]–[35]. We next treated HuCCT1 cells with the DNA-demethylating agent 5-AZA-dCR and/or the HDAC inhibitor TSA. *MiR-376c* expression was increased slightly by 5-AZA-dCR or TSA treatment alone (Figure 5C, number 4 and 5). Combination treatment with 5-AZA-dCR and TSA showed that *miR-376c* was significantly upregulated (Figure 5C, number 7 and 8). These results indicate that the synergistic effects of DNA demethylation and histone acetylation are responsible for regulation of *miR-376c* expression.

## Discussion

In the present study, we revealed that epigenetic repression of *miR-376c* accelerated EGF-dependent cell migration by targeting *GRB2* in HuCCT1 cells. The investigation of differential expression profiles between cancer and normal cells is valuable for understanding both carcinogenesis and cancer progression. We previously performed miRNA sequencing analysis of HuCCT1 and HIBEpiC [18]. *MiR-376c* was cloned exclusively from the normal cell line HIBEpiC, and not from HuCCT1. Here, we focused on functional studies of *miR-376c*, which is significantly downregulated in CC cell lines. *MiR-376c* belongs to the *miR-376* cluster gene family, containing *miR-376a*, miR-376a*, and *miR-376b*. Kawahara *et al.* showed that *miR-376* transcripts were subject to RNA editing, converting adenosine to inosine in a tissue-dependent manner, leading to a change in the silenced gene set [36]. Recently Ye *et al.* reported that activin receptor-like 7 (ALK7), which induces apoptosis via its ligand Nodal, was a target, and that it was downregulated in ovarian cancer cells [37]. Two reports of *miR-376c* upregulation in a subset of acute myeloid leukemia samples have been published [38], [39]. However, the target genes and functions of *miR-376c* in biliary epithelial cells have been not reported. *In silico* prediction of target mRNA candidates is a useful tool [40]; however, the specification of functional targets in cells by computational prediction only is generally thought to be difficult. MiRNAs are able to interact with the 3′-UTRs of many mRNAs and primarily repress their translation. Thus, we performed proteomic analysis, which is often employed to elucidate direct targets of miRNAs [41], [42]. Our results demonstrated that GRB2, an *in silico miR-376c* target candidate, was significantly downregulated in *miR-376c*-overexpressing HuCCT1 cells. Moreover, a 3′-UTR reporter assay confirmed that *miR-376c* repressed the translation of *GRB2* in a seed-sequence-dependent manner in HuCCT1 cells (Figure 2).

In migration experiments using Pre-miR-376c and siGRB2s, downregulation of GRB2 protein was shown to reduce EGF-dependent migration of HuCCT1 cells. Down-regulation of *miR-376c* allowed an increase in the amount of GRB2 protein, resulting in acceleration of HuCCT1 migration. Conversely, in normal intrahepatic biliary epithelial cells, the expression of *miR-376c* might regulate the amount of *GRB2* protein and cell migration. GRB2 is a key adapter protein, involved in signal transduction via binding to various other proteins, including the EGF receptor [24], [43], [44]. GRB2 plays essential roles in mediating EGFR signaling. A recent report showed that GRB2 interacted directly with Ras homolog family member U (RhoU) and with activated EGFR, leading to augmented JNK1 pathway activity and cell migration [45]. Although the expression and function of RhoU in bile duct cells are unknown, *miR-376c* may modulate EGF-mediated signaling through physical interaction between RhoU and GRB2. GRB2 is also well known to activate the mitogen-activated protein kinase/extracellular signal-regulated kinase pathway [46]–[48]. In cancer cells, this pathway is also activated and stimulates cell growth [47], [48]. We did not, however, observe an effect of *miR-376c* on cell growth in this cancer cell type (Figure S1).

From microarray profiling and knowledge-based network analysis, we identified IL1B and MMP9 as putative factors correlated with cell migration in HuCCT1 (Figure 4). Furthermore, in this study, we demonstrated that *IL1B* and *MMP9* were significantly downregulated in HuCCT1 cells treated with Pre-miR-376c or siGRB2. MMP9 is a matrix metallopeptidase that has been shown to be involved in degradation of basement membrane proteins in the extracellular matrix [49]. This MMP forms a homodimer, and this structure is required in the mechanism of MMP9-mediated cell migration [50]. Intriguingly, it has been reported that IL1B regulates MMP9 expression in human umbilical vein endothelial cells [51] and in extravillous trophoblasts [52]. Taken together with previous observations, our findings lend support to the idea that IL1B may be downstream of EGF signaling, such that EGF can accelerate the expression of IL1B and subsequent activation of MMP9 in HuCCT1 cells. We did not fully explore the signaling pathways of EGF-dependent cell migration in HuCCT1. Further studies will elucidate the detailed mechanisms of metastasis in ICC.

Mechanisms controlling *miR-376c* gene expression have not been reported. In this study, combination treatment with 5-AZA-dCR and TSA significantly induced the expression of *miR-376c* in HuCCT1 cells (Figure 5C), suggesting epigenetic, including histone, modification. The synergistic effect of DNA demethylation and HDAC inhibition is essential for strong expression of some genes [53], [54]. Saito *et al.* reported the miRNA expression profile of T24 bladder cancer cells treated with 5-AZA-dCR and the HDAC inhibitor 4-phenylbutyric acid, and showed that *miR-376c* was one of the miRNAs upregulated after treatment [35]. This is consistent with our results. We also found that CpG sites upstream of the *miR-376c* gene were highly methylated in HuCCT1 cells. Our data suggest that DNA methylation and HDAC modification suppress the transcription of *miR-376c* in these cells.

In summary, we have shown that *GRB2*-targeted *miR-376c* is epigenetically repressed in HuCCT1 cells, resulting in acceleration of cell migration by EGF signaling enhancement via GRB2. Since metastasis is the major cause of death in ICC, elucidating the mechanisms underlying EGF-dependent cell migration could facilitate the development of novel anti-cancer strategies for ICC using miRNAs. Although our experiments were conducted with an ICC cell line model *in vitro*, our findings on *miR-376c* function provide a new insight into pathophysiology of ICC. Further functional and pathological studies are needed to understand the mechanism[s] of miRNA-mediated posttranscriptional regulation in ICC.

## Supporting Information

Figure S1 **The effects of Pre-miR-376c and siGRB2s transfection on cell growth.** HuCCT1 cells (5×10^3^) were seeded in 96-well plates in a serum-containing medium 1 day prior to transfection. Cells were transfected with the Pre-miRNA or siGRB2s at final concentrations of 30 nM using Lipofectamine 2000 (Invitrogen) according to the manufacturer's protocol. After 24 h, the medium was changed to a medium containing RPMI 1640 supplemented with 0.1% fetal bovine serum in the presence or absence of EGF. After a further 24 h, cell growth was assayed using CellTiter-Glo (Promega, USA) according to the manufacturer's protocol. Graph shows relative cell growth. Values for cells transfected with Pre-miR-negative control (Pre-miR Cont) or nonspecific non-silencing siRNA (siRNA Cont) (AllStars Negative Control siRNA, Qiagen) were set to 1.0. No statistically significant differences between groups were detected (ANOVA followed by Tukey's test).
(TIF)

Table S1 **Genes downregulated by Pre-miR-376c and siGRB2-2.**
(XLS)

Table S2 **Genes upregulated by Pre-miR-376c and siGRB2-2.**
(XLS)

Table S3 **Molecular and cellular functions of downregulated genes associated with cellular movement in Ingenuity's Knowledge Base.**
(XLS)
