# Supplementary figures and images for: MiR-376c Down-Regulation Accelerates EGF-Dependent Migration by Targeting GRB2 in the HuCCT1 Human Intrahepatic Cholangiocarcinoma Cell Line
Source: PLoS One. 2013 Jul 26;8(7):e69496. doi: 10.1371/journal.pone.0069496 (PMC3724868; doi:10.1371/journal.pone.0069496)

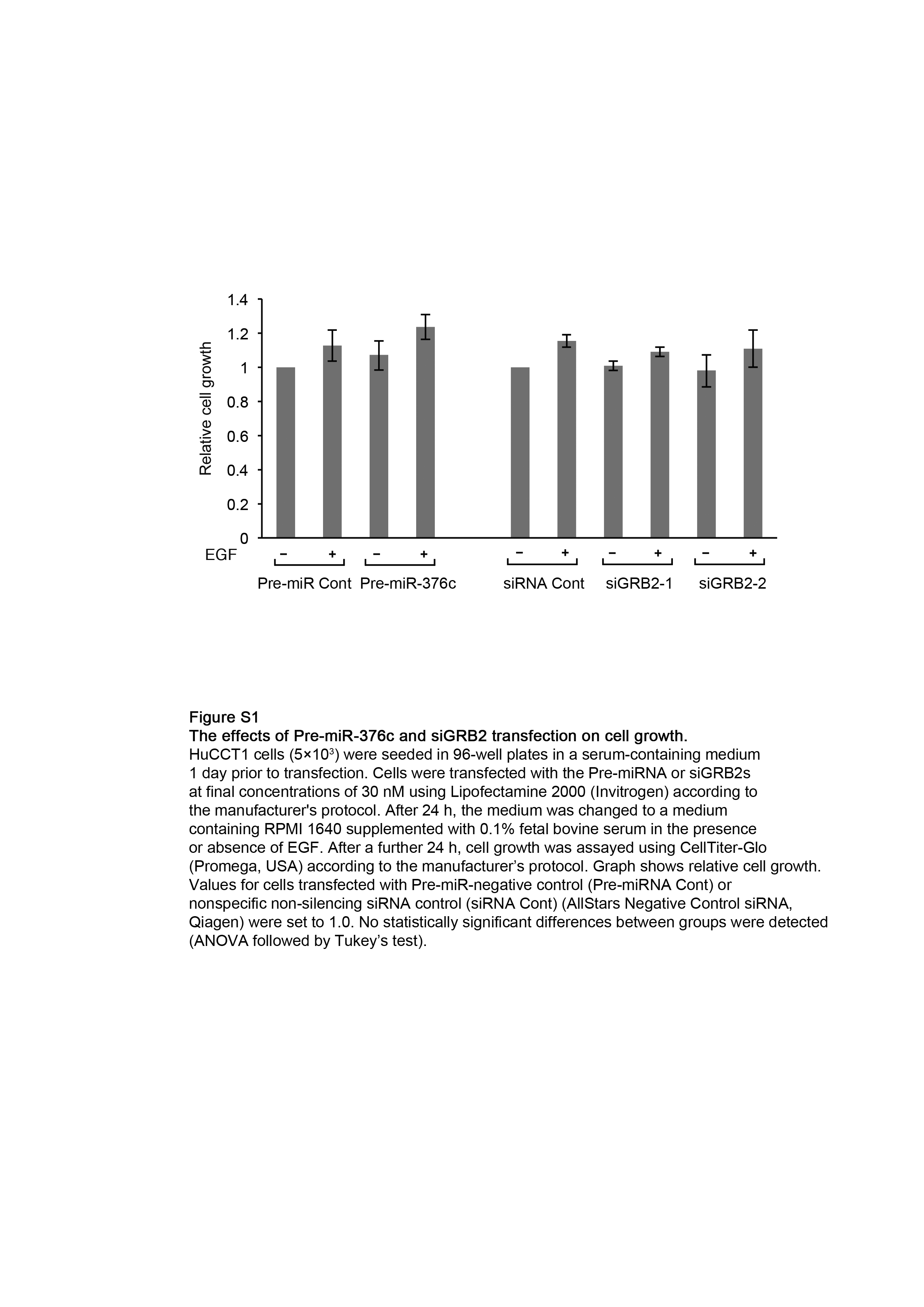

Supplement: Figure S1 — The effects of Pre-miR-376c and siGRB2s transfection on cell growth. HuCCT1 cells (5×10^3^) were seeded in 96-well plates in a serum-containing medium 1 day prior to transfection. Cells were transfected with the Pre-miRNA or siGRB2s at final concentrations of 30 nM using Lipofectamine 2000 (Invitrogen) according to the manufacturer's protocol. After 24 h, the medium was changed to a medium containing RPMI 1640 supplemented with 0.1% fetal bovine serum in the presence or absence of EGF. After a further 24 h, cell growth was assayed using CellTiter-Glo (Promega, USA) according to the manufacturer's protocol. Graph shows relative cell growth. Values for cells transfected with Pre-miR-negative control (Pre-miR Cont) or nonspecific non-silencing siRNA (siRNA Cont) (AllStars Negative Control siRNA, Qiagen) were set to 1.0. No statistically significant differences between groups were detected (ANOVA followed by Tukey's test). (TIF) [file pone.0069496.s001.tif]
